# Multiclass Machine Learning-Based Discovery of Novel Scaffold Inhibitors Targeting ALK

**DOI:** 10.3390/ph19081209

**Published:** 2026-08-01

**Authors:** Md Azizul Haque, Qazi Mohammad Sajid Jamal, Khurshid Ahmad, Reem Binsuwaidan, Nawaf Alshammari, Mohd Saeed, Jong-Joo Kim, Danishuddin

**Affiliations:** 1 Department of Biotechnology, Yeungnam University, Gyeongsan 38541, Republic of Korea; azizul@ynu.ac.kr; 2 Department of Health Informatics, College of Applied Medical Sciences, Qassim University, Buraydah 51452, Saudi Arabia; m.quazi@qu.edu.sa (Q.M.S.J.); k.ahmad@qu.edu.sa (K.A.); 3 Department of Pharmaceutical Sciences, College of Pharmacy, Princess Nourah Bint Abdulrahman University, Riyadh 11671, Saudi Arabia; rabinsuwaidan@pnu.edu.sa; 4 Department of Biology, College of Sciences, University of Hail, Hail 55476, Saudi Arabia; naib.alshammari@uoh.edu.sa (N.A.); mo.saeed@uoh.edu.sa (M.S.)

**Keywords:** ALK inhibitors, multiclass modeling, machine learning, molecular descriptors, fingerprints, novel scaffolds

## Abstract

**Background**: Anaplastic Lymphoma Kinase (ALK) is an oncogenic receptor tyrosine kinase implicated in several cancers. Despite the clinical success of ALK inhibitors, acquired resistance continues to drive the search for novel chemotypes. We developed a multiclass machine learning framework to classify ALK inhibitory activity using a curated ChEMBL dataset. **Methods**: Models were built using 2D molecular descriptors together with MACCS and ECFP4 fingerprints. Three widely used algorithms, Support Vector Machine (SVM), Random Forest (RF), and XGBoost, were applied for model development. **Results**: RF and XGBoost models demonstrated the best performance, achieving accuracies of ~0.75–0.79 with consistently high ROC–AUC values, particularly for fingerprint-based features. Bemis–Murcko scaffold analysis identified enriched chemotypes and underexplored scaffolds for further prioritization. The validated models were subsequently used to screen the Maybridge library, and compounds predicted to possess potential ALK inhibitory activity were prioritized for further computational evaluation. Applicability-domain filtering confirmed that the selected compounds occupied the predicted ALK inhibitor chemical space across multiple activity classes. The shortlisted compounds were subsequently evaluated by molecular docking to characterize their binding modes and interactions. Three candidate hits (SCR00078, SCR00073, and AW01085) were selected for further evaluation using 500 ns molecular dynamics simulations alongside the reference inhibitor Brigatinib. Simulation analyses revealed stable protein–ligand complexes and reduced conformational fluctuations relative to apo ALK, while MM/PBSA calculations identified SCR00078 and AW01085 as the most favorable binders. **Conclusions**: This integrated ML-to-simulation workflow prioritizes structurally novel candidate hits with predicted ALK inhibitory activity and provides an effective strategy for scaffold discovery and hit prioritization.

## 1. Introduction

Anaplastic Lymphoma Kinase (ALK) is a receptor tyrosine kinase encoded by the ALK gene located on chromosome 2p23 [1]. It is a crucial component in cellular signaling networks and is highly expressed during embryogenesis, particularly in the nervous system, where it regulates neuronal differentiation, synaptic plasticity, and overall brain development [2]. ALK functions as a transmembrane receptor that is activated by ligand binding, leading to dimerization and autophosphorylation, triggering downstream signaling cascades. These pathways, including MAPK/ERK, PI3K/AKT, and JAK/STAT, regulate cell proliferation, survival, differentiation, and migration [3]. The tightly regulated activity of ALK is essential for normal development; however, aberrant ALK activity due to mutations, gene rearrangements, or amplifications can lead to uncontrolled cell growth and malignancy, underscoring its importance as a therapeutic target [4]. ALK gene rearrangements were first identified in anaplastic large cell lymphoma (ALCL) as the NPM-ALK fusion and have since been observed in several cancers, including non-small cell lung cancer (NSCLC), neuroblastoma, and inflammatory myofibroblastic tumors [5]. In NSCLC, approximately 3–7% of patients harbor ALK fusions, with EML4-ALK being the most common variant [6]. These fusion proteins constitutively activate ALK activity, bypassing normal ligand-mediated regulatory mechanisms. As a result, oncogenic signaling pathways are continuously active, driving tumorigenesis by promoting proliferation, survival, and metastasis [7]. Additionally, ALK fusions are associated with distinct clinicopathological features, including younger patient age, non-smoker status, and adenocarcinoma histology, making ALK not only a therapeutic target but also a biomarker for patient stratification [8].

The development of ALK inhibitors has transformed the therapeutic landscape for ALK-positive cancers. Crizotinib, a first-generation ALK inhibitor, was the first targeted therapy approved for ALK-positive NSCLC, demonstrating remarkable efficacy in objective response rates and progression-free survival compared with standard chemotherapy [9]. However, despite initial responses, patients frequently develop acquired resistance to crizotinib due to secondary mutations in the ALK domain, such as L1196M, C1156Y, and G1269A, or to activation of bypass signaling pathways, such as EGFR, KIT, and KRAS [10]. To overcome these resistance mechanisms, second-generation inhibitors such as ceritinib, alectinib, and brigatinib were developed. These inhibitors exhibit greater potency, improved central nervous system penetration, and activity against a broader spectrum of resistance mutations [11]. Despite these advances, resistance remains a significant clinical challenge, particularly in the presence of mutations such as G1202R, which confers resistance to multiple second-generation inhibitors [7]. These challenges underscore the need for new therapeutic strategies that target ALK through distinct mechanisms, reduce off-target effects, and overcome existing resistance pathways.

In recent years, computational methods have emerged as powerful tools in drug discovery, particularly in the design and identification of novel kinase inhibitors [12]. Quantitative structure–activity relationship (QSAR) models are computational approaches that correlate chemical structural features with biological activity, enabling the prediction of potential inhibitors before experimental validation. Machine learning techniques further enhance QSAR by identifying complex, non-linear relationships between molecular descriptors and activity profiles, thereby allowing high-throughput screening of chemical libraries to identify promising candidates [13]. Multiclass QSAR models, which categorize compounds into multiple activity classes rather than a simple binary active/inactive classification, provide a more nuanced understanding of structure–activity relationships, particularly when studying compounds with variable inhibitory potency or resistance profiles [14]. The integration of QSAR, cheminformatics, and molecular docking enables a rational, hypothesis-driven approach to drug discovery. For ALK inhibitors, this approach can identify chemical scaffolds that are more likely to retain efficacy against resistant mutations, optimize binding affinity, and minimize toxicity. Previous studies have utilized QSAR and pharmacophore modeling to design inhibitors targeting ALK and other tyrosine kinases, demonstrating the potential of these approaches to accelerate drug development and reduce experimental costs. To further enhance compound prioritization, machine learning algorithms were employed as predictive screening tools within the virtual screening workflow. These approaches enable the identification of compounds with a higher likelihood of ALK inhibitory activity prior to downstream structure-based analyses (Supplementary Figure S1). This study focuses on developing robust multiclass QSAR models to predict the inhibitory activity of novel ALK inhibitors. Using a diverse dataset of known inhibitors and non-inhibitors, molecular descriptors and fingerprints are calculated to capture structural, electronic, and physicochemical features relevant to ALK binding. Various machine learning algorithms, including random forest, support vector machines (SVM), and gradient boosting, are employed to optimize model performance, with cross-validation and external validation ensuring predictive reliability.

## 2. Results

### 2.1. Machine Learning Model Performance

**Figure 1:**
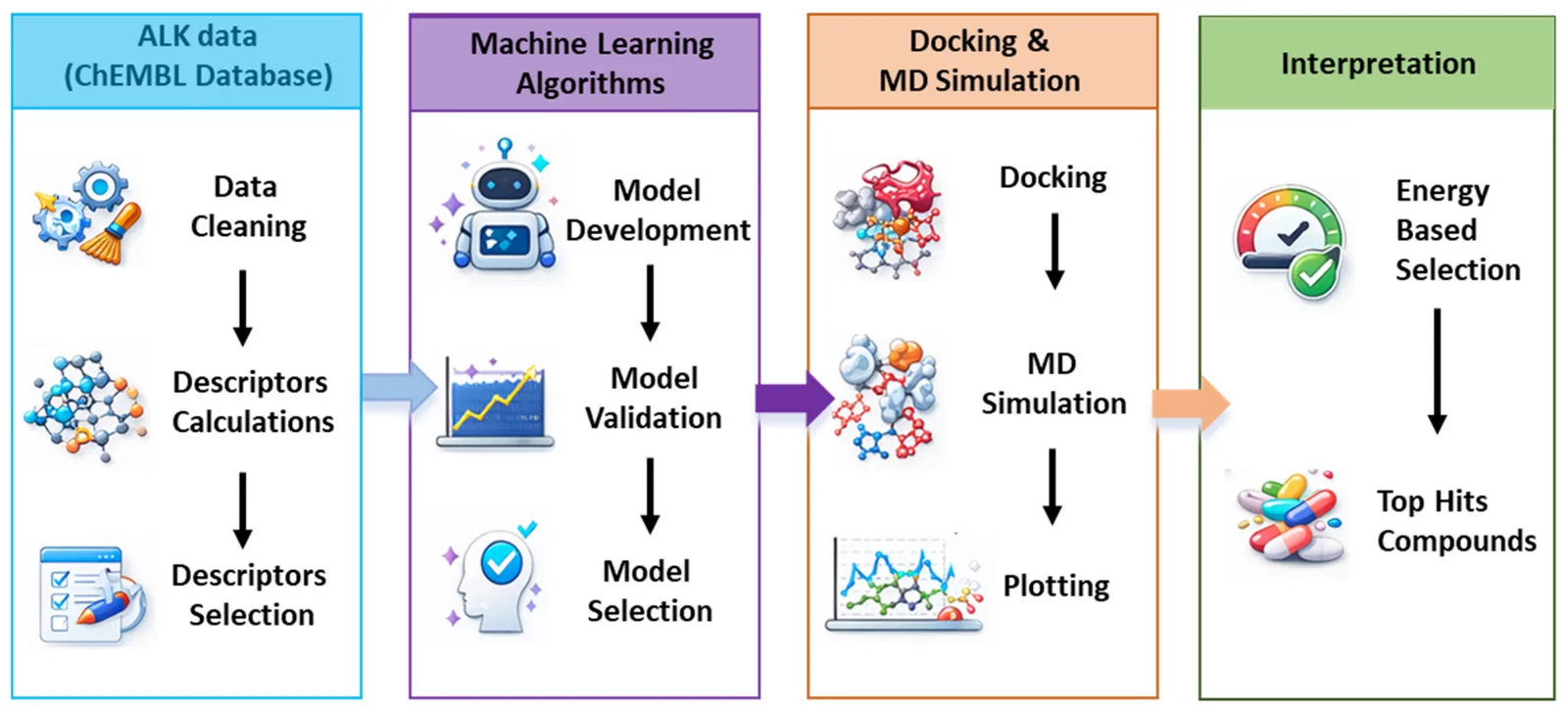
Computational workflow for identification and prioritization of candidate ALK inhibitors. ChEMBL-derived ALK data were processed and used to train machine learning models (RF, SVM, XGBoost). Predicted hits were evaluated by molecular docking and molecular dynamics simulations, followed by interpretation of binding interactions and stability to identify promising inhibitors.

**Figure 2:**
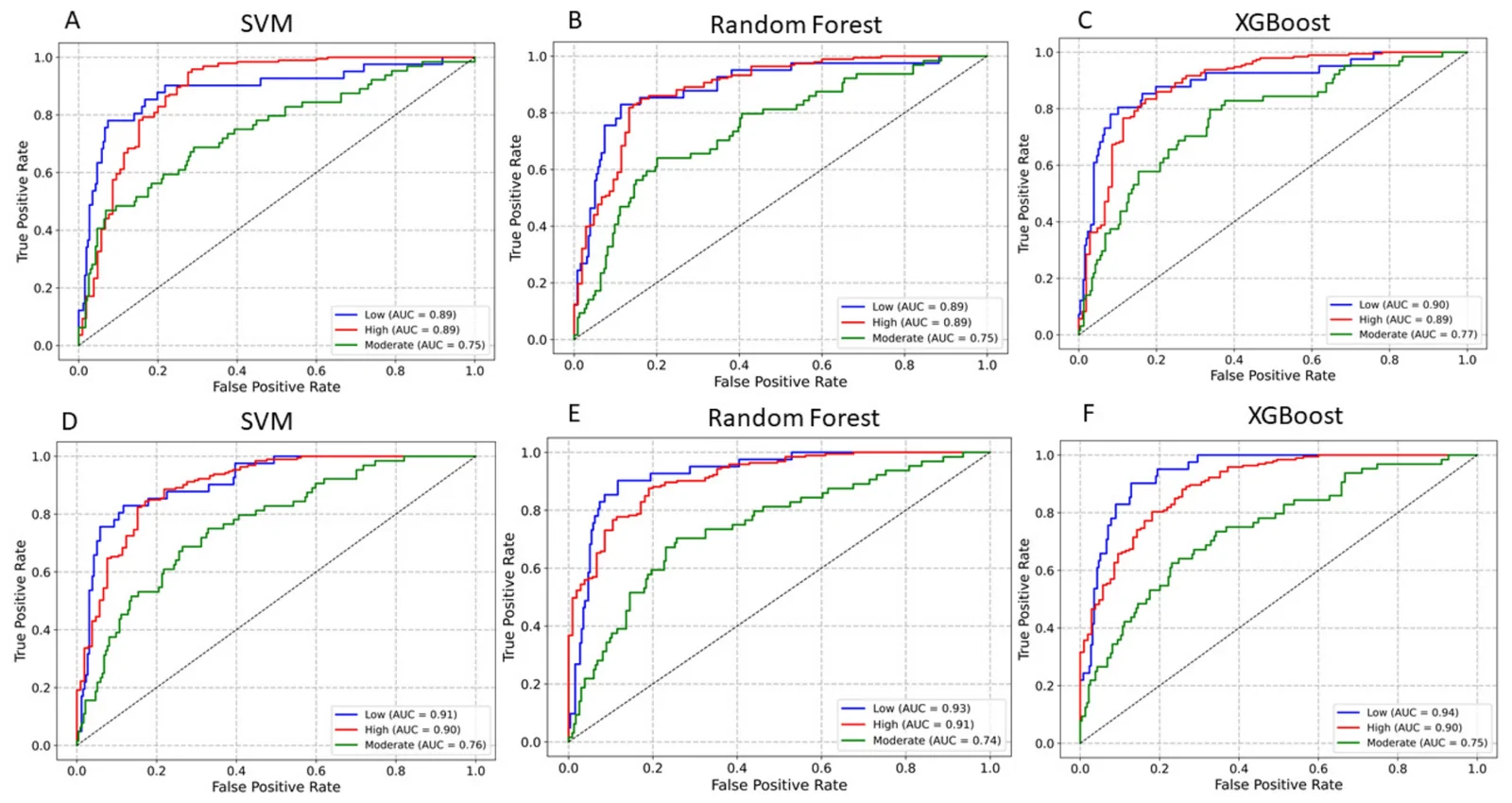
ROC curve showing the performance of multiclass models. (**A**–**C**) 2D descriptors set. (**D**–**F**) Fingerprints.

The workflow used to perform the study is shown in Figure 1. Table 1 summarizes the predictive performance of the SVM, RF, and XGB models developed using two different molecular representations: 2D descriptors and molecular fingerprints (MACCS and ECFP4). All three models demonstrated satisfactory predictive ability; however, model performance varied depending on the descriptor type. Using 2D descriptors, the SVM model achieved the highest classification accuracy (0.79) with balanced precision and recall values across activity classes. RF and XGB also showed strong predictive performance, although with slightly lower overall accuracy. Nevertheless, XGB exhibited comparatively higher AUC values for the high- and low-activity classes, indicating better discrimination for extreme activity categories. Models developed using molecular fingerprints generally outperformed those based on 2D descriptors, suggesting that fingerprint representations captured richer structural and substructural information relevant to ALK inhibitory activity. Among these models, RF achieved the best overall performance, with an accuracy of 0.78 and AUC values ranging from 0.91 to 0.93. XGB showed comparable predictive ability and achieved the highest AUC for the low-activity class (0.94). SVM also maintained good predictive performance but remained slightly inferior to the ensemble-based RF and XGB models. Across all models, the high- and low-activity classes were classified more accurately than the moderate-activity class. The AUC values for high- and low-activity compounds ranged from approximately 0.89 to 0.94, whereas moderate-activity compounds showed lower AUC values (~0.74–0.77). Figure 2 illustrates the ROC curves obtained for all models using both descriptors sets. All curves were positioned substantially above the random classification line, confirming that the models performed significantly better than random prediction. RF and XGB exhibited consistently high and stable AUC values for both training and test datasets, indicating robust generalization performance without substantial overfitting. SVM also showed reliable performance, although slightly lower than the ensemble models.

### 2.2. Chemical Space Analysis

**Figure 3:**
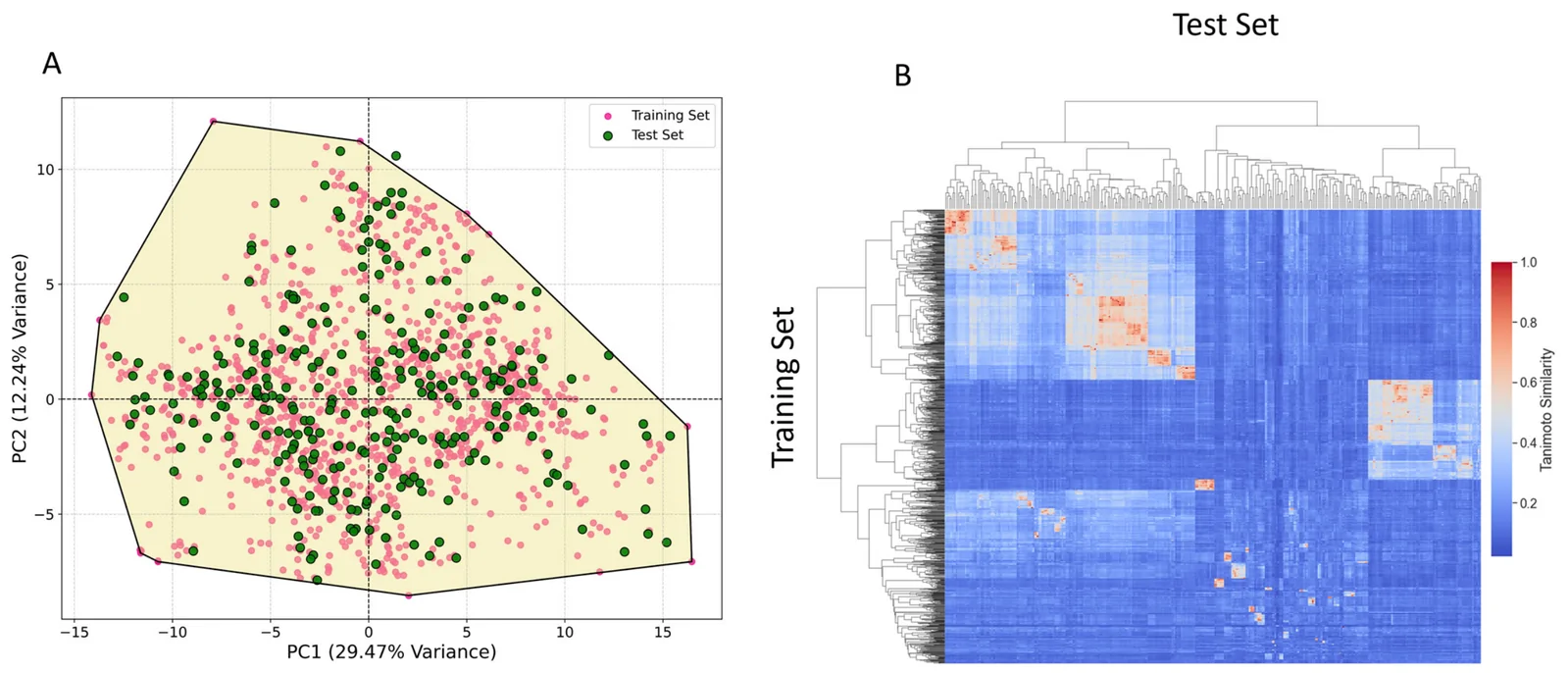
Chemical space analysis of the training and test datasets. (**A**) PCA score plot showing the distribution of the training set and test set compounds within the defined chemical space. (**B**) Hierarchical clustering heatmap of pairwise Tanimoto similarity, demonstrating that the test set is well represented within the training set chemical space while retaining structural diversity.

The chemical space distribution of the training and test sets was evaluated using principal component analysis (PCA) and pairwise Tanimoto similarity heatmap analysis (Figure 3A,B). PCA was employed to visualize the distribution of compounds in descriptor space and to assess the overlap between datasets. The first two principal components explained 29.48% and 12.41% of the total variance, respectively. As shown in Figure 3A, the training and test compounds exhibited substantial overlap in the PCA space, indicating that the test set compounds are within the chemical space of the training set. The broad distribution of compounds across the PCA space shows the structural diversity of the dataset, while the observed overlap between the training and test sets demonstrates adequate chemical space coverage. Figure 3B presents the pairwise Tanimoto similarity matrix, where each cell represents the structural similarity between two compounds. Hierarchical clustering revealed localized regions of higher similarity corresponding to shared scaffolds or chemotypes, whereas the predominance of lower similarity values indicates substantial structural diversity within the dataset. Together, the PCA and similarity heatmap analyses demonstrate that the dataset combines broad chemical diversity with adequate chemical space coverage, supporting the development of robust and generalizable predictive models.

### 2.3. Y-Scrambling Validation

**Figure 4:**
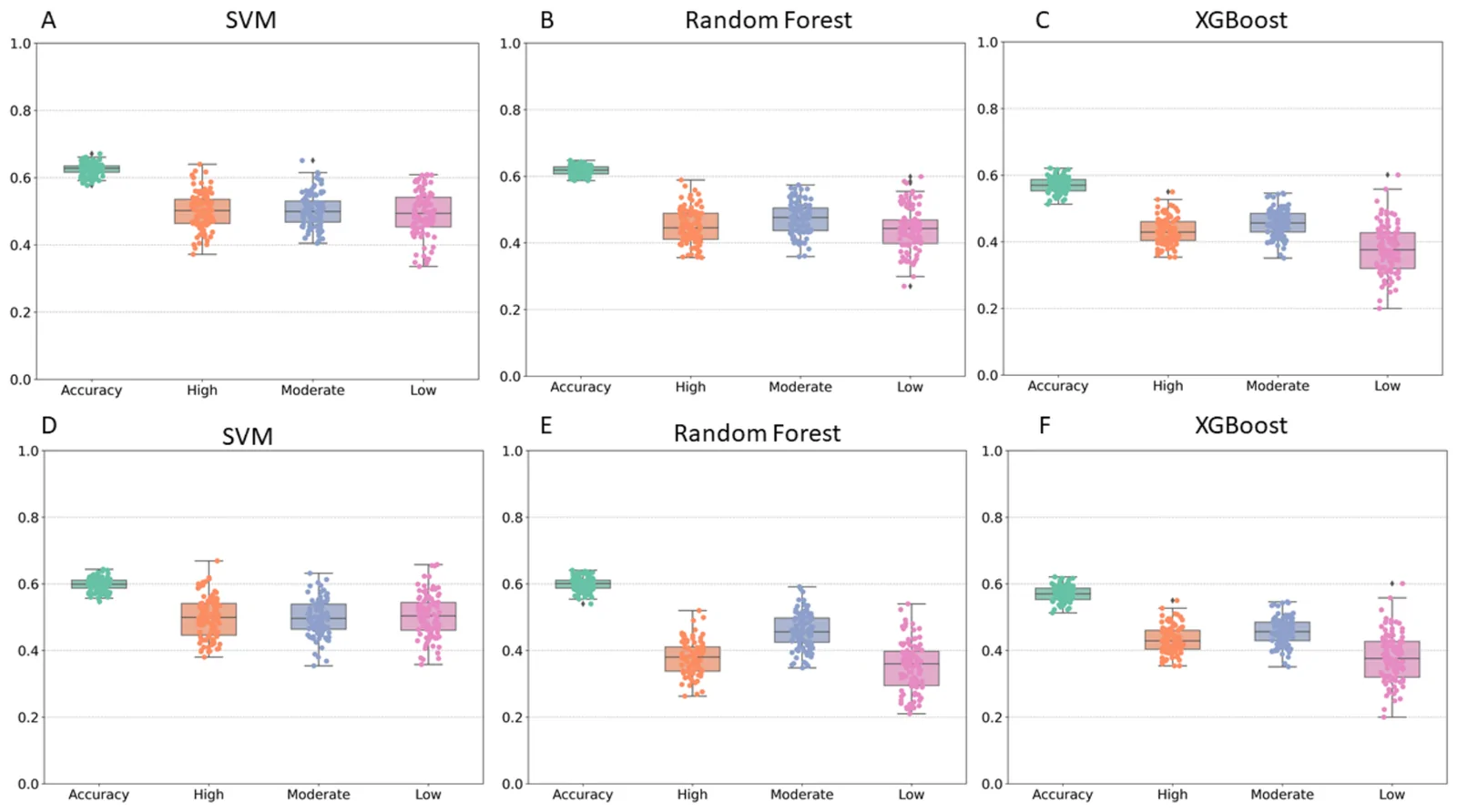
Box plot showing the frequencies of accuracies from Y-randomization models. (**A**–**C**) 2D Descriptors. (**D**–**F**) Fingerprints. Total of 100 Y-randomization runs were performed.

Figure 4 presents the Y-scrambling results for the three machine learning models, SVM, RF, and XGB, to evaluate the robustness and reliability of their predictive performance on the models. In this validation procedure, the activity labels were randomly shuffled multiple times while the molecular descriptors remained unchanged, and the models were retrained at each iteration. This process tests whether the original SAR learned by the models is genuine or a result of random correlations. Across all models and descriptor sets, the boxplots show that both accuracy and ROC-AUC values for the High, Moderate, and Low activity classes fall within a narrow, low range (approximately 0.3–0.6), indicating that when the response variable is randomized, the models lose their predictive ability (Figure 4A–C). The corresponding quantitative performance metrics are summarized in Table 2, which consistently shows low accuracy, precision, F1-score, and ROC-AUC values across all models and descriptor sets following label randomization. This sharp decline compared to the high performance observed in the original, non-scrambled models confirms that their predictive power is not due to chance. A consistent difference in ROC-AUC and class-wise performance is observed when compared with the original models, indicating the sensitivity of model predictions to label permutation (Table 2). Although some Y-scrambled models retained moderate accuracy values, their ROC-AUC, precision, and F1-scores decreased substantially and approached values expected for random classification, indicating a marked loss of discriminatory ability after label permutation (Table 2). Comparing the two descriptor types, both 2D descriptors and fingerprints yield similar Y-scrambled results, showing no significant spurious correlations in either feature set. However, models trained on fingerprints (Figure 4D–F) show slightly higher variability, likely reflecting the higher dimensionality and complexity of these fingerprints. Overall, the Y-scrambling validation demonstrates that the SVM, RF, and XGB models capture genuine and meaningful relationships between molecular structure and biological activity. The consistent drop in performance upon label permutation confirms that the models’ success in predicting compound activity is statistically significant and not due to random noise, thereby reinforcing their robustness and reliability for virtual screening and compound prioritization.

### 2.4. SHAP Analysis

**Figure 5:**
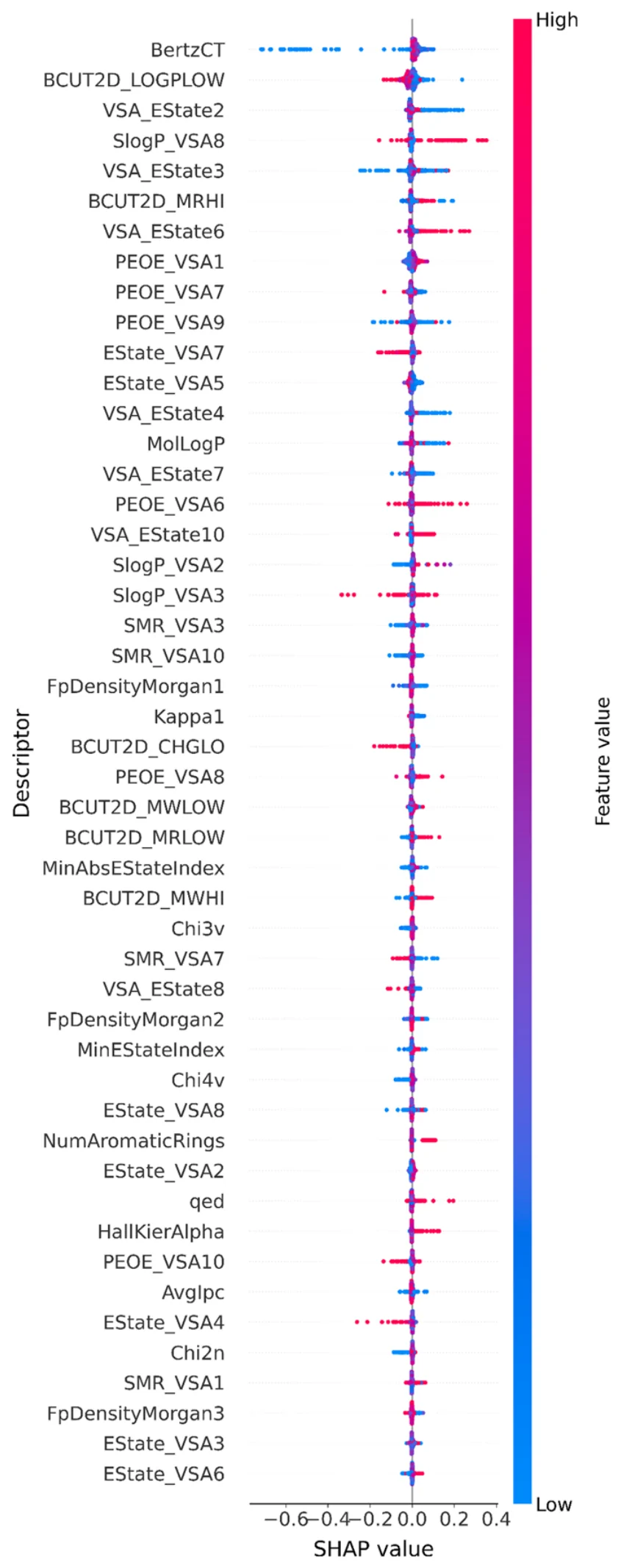
SHAP (SHapley Additive exPlanations) summary plot showing the impact of 2D molecular descriptors on the predictive output of the multiclass ALK inhibitor model. Each point represents a feature value for a single compound, with color indicating the feature value from low (blue) to high (red). Features are ranked by their overall importance in influencing the model, with positive SHAP values driving predictions toward higher activity and negative values toward lower activity.

The SHAP summary plot (Figure 5) was used to evaluate the contribution of selected molecular descriptors toward predicting ALK inhibitory activity. Among the descriptors, BertzCT, BCUT2D_LOGPLOW, VSA_EState2, SlogP_VSA8, BCUT2D_MRHI, and PEOE_VSA1 emerged as the most influential features. These descriptors collectively describe molecular complexity, lipophilicity, electronic distribution, molecular refractivity, and surface area properties. Descriptors associated with lipophilicity and surface electronic characteristics contributed strongly toward predicting highly active ALK inhibitors, emphasizing the importance of hydrophobic and electrostatic interactions within the ATP-binding pocket of ALK. BCUT2D descriptors further highlighted the role of balanced charge distribution, polarizability, and refractive properties in ligand recognition. In addition, the significance of BertzCT suggested that moderate molecular complexity favored efficient kinase recognition while minimizing steric hindrance. The SHAP analysis therefore demonstrated that the machine learning model successfully identified chemically meaningful descriptors consistent with experimentally established SAR characteristics of ALK inhibitors.

### 2.5. Scaffold Enrichment and SAR Complexity

**Figure 6:**
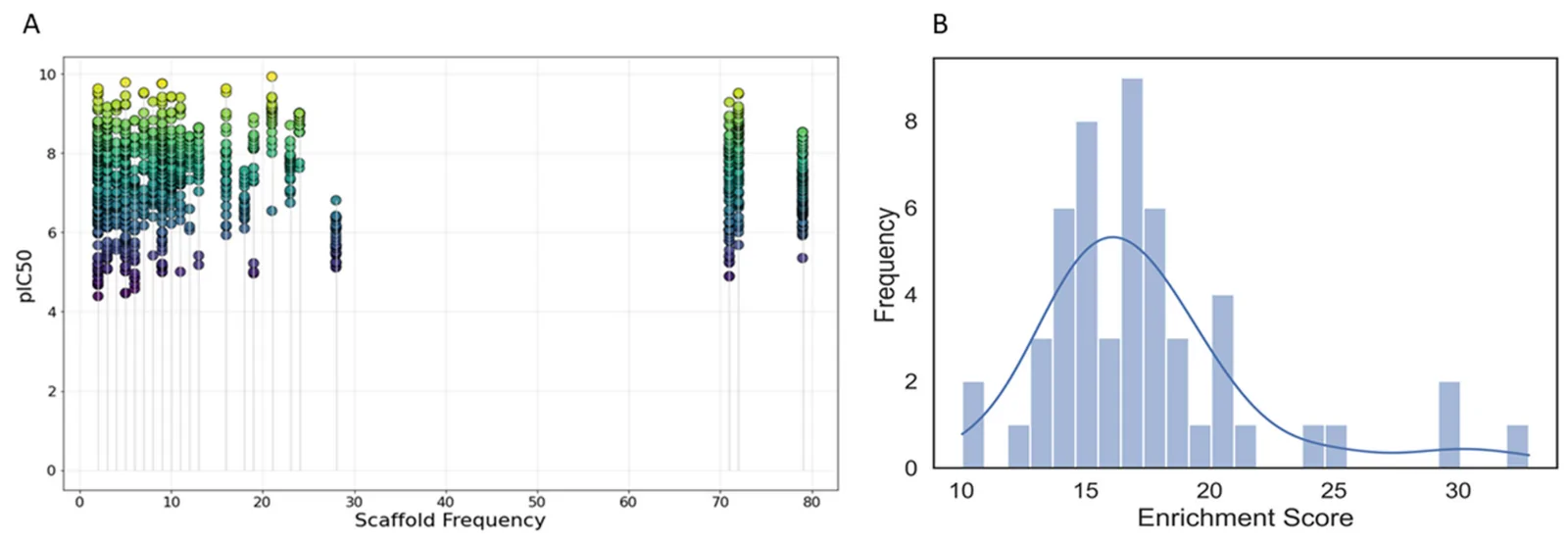
Scaffold activity and enrichment analysis. (**A**) Relationship between scaffold frequency (log scale) and mean pIC_50_, with point size representing activity variability. (**B**) Distribution of scaffold enrichment scores, showing the frequency and density of enriched chemical scaffolds within the dataset.

Figure 6 presents a comprehensive scaffold-centric analysis that elucidates the interplay between chemical representation, biological potency, and structure–activity relationship (SAR) complexity within the dataset. As shown in Figure 6A, scaffold frequencies span a broad range from sparsely represented to highly populated chemotypes; however, mean pIC_50_ values do not exhibit a direct dependence on scaffold frequency. Several low-frequency scaffolds demonstrated potency comparable to or greater than highly represented scaffolds, indicating the presence of underexplored chemotypes with potential pharmacological significance. The substantial vertical spread observed within individual scaffold frequency groups further suggested pronounced intra-scaffold SAR variability, highlighting the importance of substituent-level modifications in modulating ALK inhibitory activity. The scaffold enrichment score distribution exhibited a right-skewed profile (Figure 6B), indicating that only a limited subset of scaffolds simultaneously combined high potency with sufficient structural representation to emerge as dominant contributors within the dataset.

### 2.6. Chemical Screening and Molecular Docking

**Figure 7:**
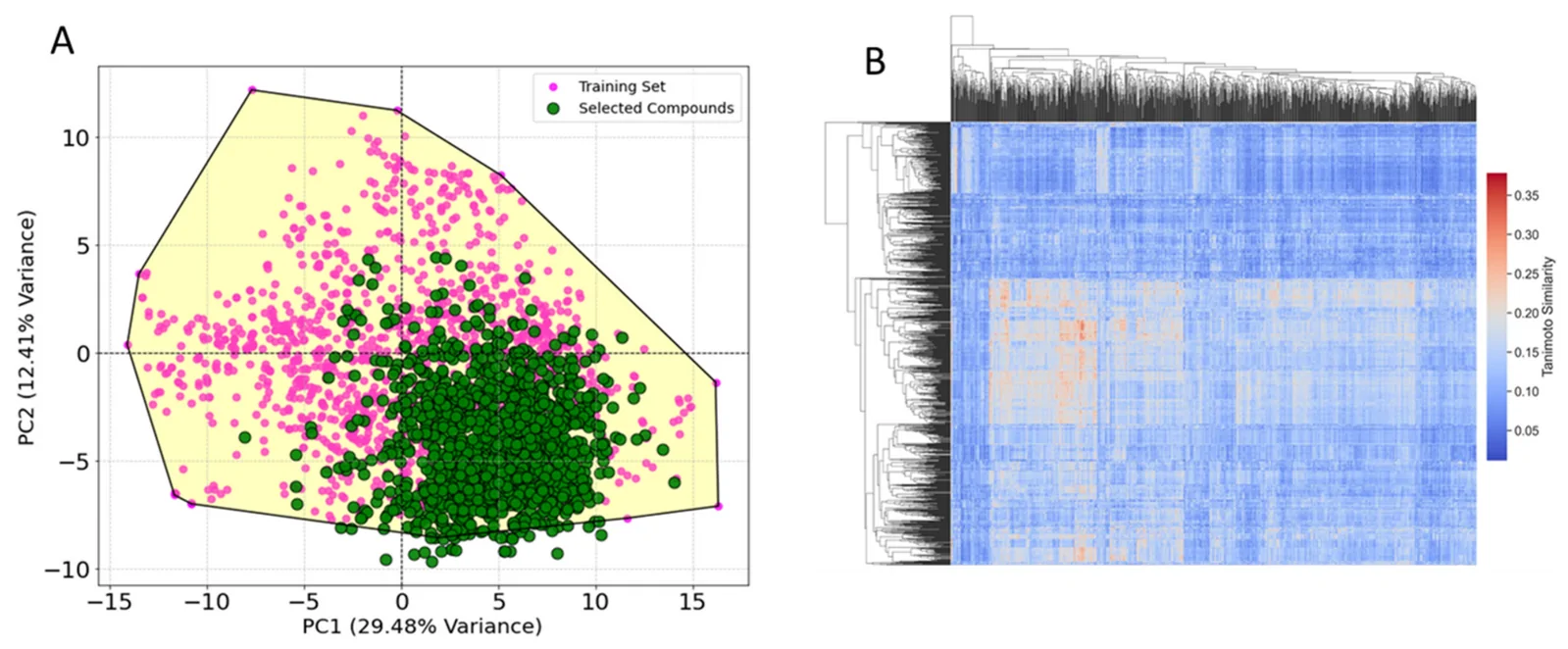
Library screening result. (**A**) Chemical space of selected active hits. (**B**) Heatmap showing the Tanimoto similarity score (Tc) of selected compounds with hits and training set compounds. ECFP4 fingerprints were used to calculate the Tanimoto coefficient similarities.

Maybridge Library has been used to screen hits from validated models (Figure 7). Compounds predicted as active by the validated model were selected and filtered based on the training set’s applicability domain (Figure 7). A total of 1094 compounds met the applicability criteria (AD) and were further subjected to molecular docking-based virtual screening. The top 3 compounds from docking-based screening were further processed for MD simulation.

### 2.7. Molecular Docking Analysis

**Figure 8:**
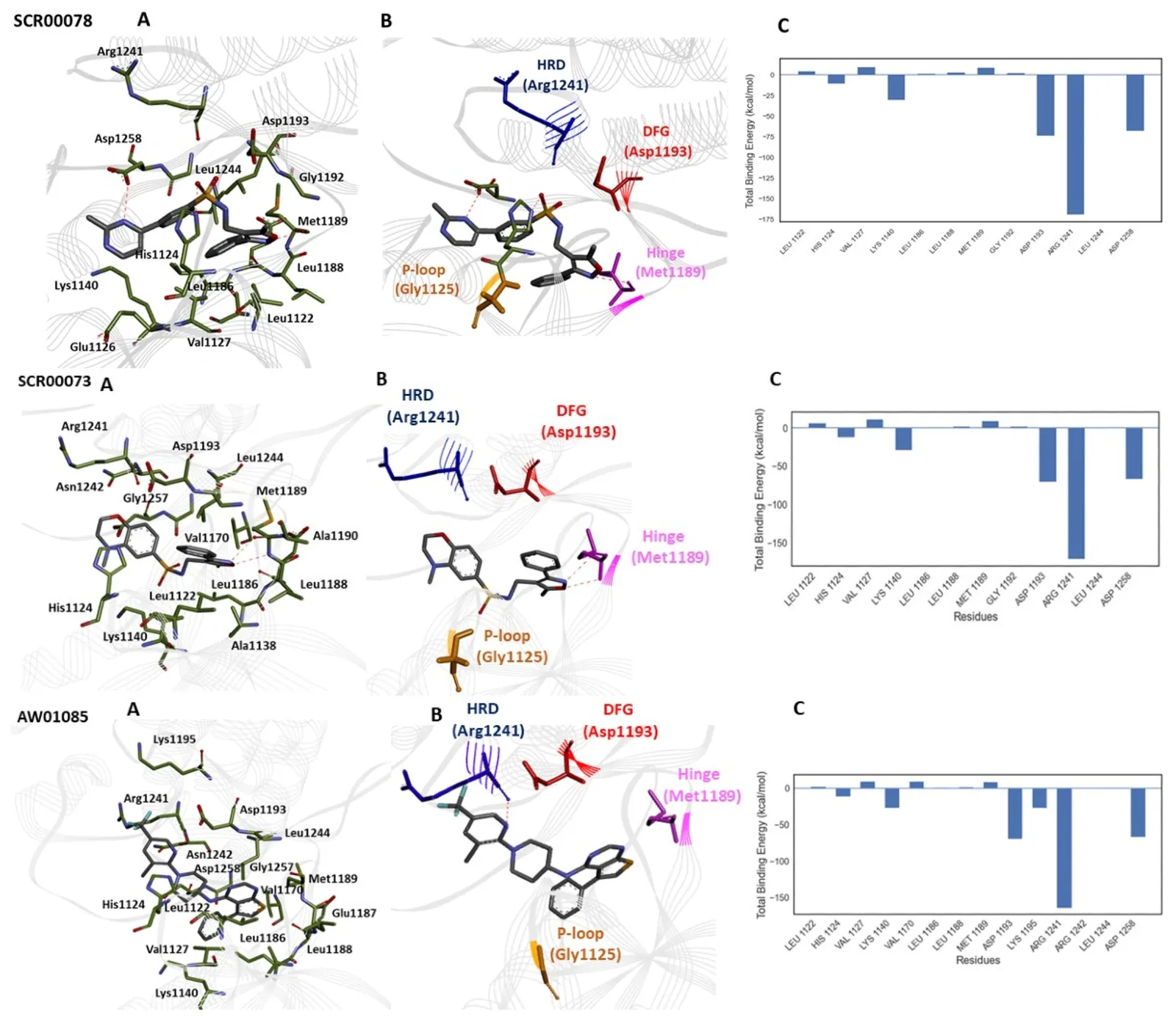
Predicted binding modes of the selected compounds in the ALK binding site. (**A**) Three-dimensional binding poses of SCR00078, SCR00073, and AW01085 within the ALK active site. (**B**) Key interactions with conserved kinase motifs, including the hinge region (Met1189), DFG motif (Asp1193), HRD motif (Arg1241), and glycine-rich P-loop. (**C**) Binding energy contributions of key binding-site residues.

Docking analyses show that all selected compounds (SCR00078, SCR00073 and AW01085) interacted favorably with key functional regions of the ALK ATP-binding pocket, including the hinge region, hydrophobic core, glycine-rich P-loop, and activation loop (Figure 8). Compound SCR00078, with a binding energy of −8.2 kcal/mol, adopts a compact conformation within the ALK ATP-binding pocket, stabilized predominantly by hydrophobic interactions. It is well accommodated within a lipophilic core formed by Leu1122, Ala1138, Val1170, Leu1186, and Leu1188, which closely surround its aromatic scaffold (Figure 8). Compound SCR00073 displays an extended binding mode, enabling the engagement of both hydrophobic and polar regions. However, this compound exhibits a binding energy of −8.2 kcal/mol, which is comparable to that of SCR00078. It maintains strong hydrophobic interactions with Leu1122, Val1170, Leu1186, and Leu1188 (Figure 8), consistent with reported binding modes of ALK inhibitors. Met1189 forms a stabilizing hydrogen bond, reinforcing its anchoring role. Interactions with Asp1193 and Arg1241 provide additional electrostatic stabilization, while contacts with Gly1125 and His1124 further contribute to stabilization of the binding pose. Furthermore, interactions with Asp1193 suggest involvement of the activation loop (A-loop/DFG motif region). Compound AW01085 adopts a distinct orientation that allows interaction with both the hydrophobic core and the solvent-accessible region. It exhibits a binding energy of −8.4 kcal/mol against ALK. The ligand maintains key hydrophobic contacts with Leu1122, Val1130, Val1170, Leu1186, and Leu1188, ensuring stable core binding (Figure 8). In addition, strong interactions with Asp1193 and Arg1241 highlight the importance of electrostatic contributions in stabilizing the complex. Similar to compound SCR00073, interactions with Asp1193 further support engagement with the activation loop region, reinforcing conformational stabilization of the kinase domain. Energy decomposition analysis confirms that, while van der Waals interactions remain significant, electrostatic contributions, particularly from Arg1241, play a prominent role, providing a complementary and well-supported binding mechanism. Overall, all three compounds demonstrate consistent engagement with critical ALK residues, particularly Met1189 (hydrogen bonding), hydrophobic Leu/Val residues (core stabilization), and Asp1193/Arg1241 (electrostatic interactions). Collectively, these interactions span key functional regions of ALK, including the hinge region, glycine-rich P-loop, and activation loop, which are essential determinants of kinase inhibition. Overall, the three compounds exhibited comparable docking scores but distinct binding orientations within the ALK ATP-binding pocket. Despite these differences, all complexes retained interactions with key functional regions of the kinase, including the hinge region and residues surrounding the activation loop, suggesting that multiple binding geometries may support stable ATP-pocket recognition. Considering the small differences in predicted docking energies, molecular docking was used primarily to identify plausible binding modes and key protein–ligand interactions rather than to establish superiority among the compounds.

### 2.8. Physicochemical and ADMET Profiling of Selected Hits

To complement docking and simulation-based prioritization, the physicochemical and ADMET profiles of the three selected hits were evaluated. A compact consensus summary is provided in Table 3, highlighting drug-likeness compliance, absorption/distribution indicators, key CYP-related liabilities, and major safety flags predicted in silico. Overall, the three hits showed acceptable physicochemical ranges compatible with oral drug-like space, with no pan-assay interference (PAINS) alerts and minimal structural alert flags. However, in silico safety profiling flagged potential liabilities in selected endpoints (notably cardiotoxicity risk via hERG prediction and/or hepatotoxicity propensity in some models), indicating that future hit-to-lead optimization should explicitly address these risks during scaffold refinement. Radar plots are qualitative and intended for comparative visualization of model outputs.

### 2.9. Conformational Stability and Dynamics from MD Simulations

**Figure 9:**
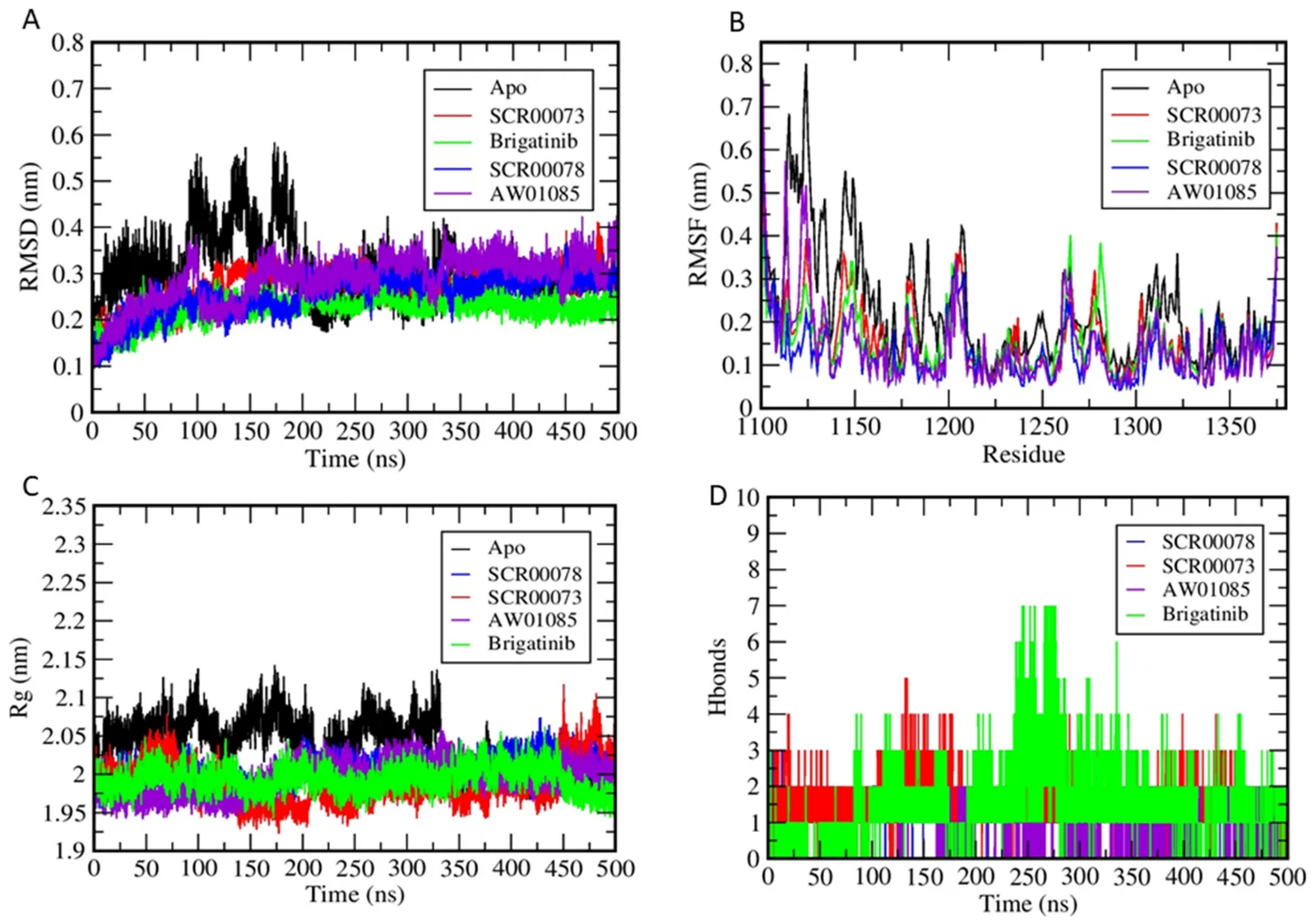
Molecular dynamics simulation analysis of ALK systems. (**A**) RMSD of backbone atoms as a function of simulation time (0–500 ns), indicating the structural stability of the apo protein and ligand-bound complexes. (**B**) RMSF per residue, highlighting residue-level flexibility across the protein sequence. (**C**) Rg profiles showing the overall compactness and folding stability of the systems during the simulation. (**D**) Number of intermolecular hydrogen bonds (H-bonds) formed between the protein and ligands over the simulation time.

The molecular dynamics trajectories provide not only an overall assessment of structural stability but also reveal critical time-dependent conformational transitions relevant to ALK’s functional dynamics. The RMSD profile of the apo system shows a pronounced increase between ~80–200 ns, reaching ~0.5–0.6 nm, which likely reflects a major conformational rearrangement associated with activation loop (A-loop) movement or partial opening of the kinase domain (Figure 9). After ~200 ns, although partial stabilization is observed, the persistently elevated RMSD suggests that the apo protein continues to sample multiple conformational states in the absence of ligand-imposed constraints. In contrast, ligand-bound systems equilibrate earlier (typically ~100–150 ns), after which RMSD values plateau, indicating enhanced structural stability. Brigatinib, in particular, rapidly stabilized within ~100 ns and maintained minimal deviation throughout the simulation, suggesting effective restriction of conformational mobility upon binding. The compounds (SCR00073, SCR00078, and AW01085) also stabilize but exhibit comparatively broader RMSD distributions, suggesting weaker conformational constraints (Figure 9). Minor fluctuations observed in later stages (~300–400 ns), especially for SCR00073, are likely attributable to localized adjustments within the binding pocket rather than global structural rearrangements. The RMSF analysis further highlights functionally important flexible regions, particularly residues ~1110–1150, where the apo system exhibits the highest fluctuations (Figure 9). These regions likely correspond to regulatory loop elements proximal to the ATP-binding site, including the A-loop or P-loop, which play essential roles in substrate accessibility and kinase activation. The marked reduction in RMSF upon ligand binding indicates that these compounds effectively stabilize key dynamic regions. Brigatinib demonstrates the strongest suppression of residue-level fluctuations, suggesting efficient locking of the kinase in a stable, more conformationally constrained state. From a compactness perspective, the radius of gyration (Rg) profile supports these observations, with the apo system showing notable fluctuations during ~100–250 ns (Figure 9), consistent with structural rearrangements. After this period, the Rg stabilizes but remains slightly higher than in ligand-bound systems, reflecting a less compact and more flexible structure. In contrast, ligand-bound complexes, including Brigatinib and the screened compounds (SCR00073, SCR00078 and AW01085), maintain relatively stable Rg profiles throughout the simulation, with only minor deviations at later time points (~300–500 ns). Notably, SCR00078 and AW01085 exhibit Rg stability comparable to Brigatinib over most of the simulation, indicating effective maintenance of protein compactness. Although SCR00073 shows a slight increase in Rg near 450 ns, this may reflect a localized conformational adjustment rather than global destabilization, suggesting that the overall structural integrity of the complex is preserved. Hydrogen bond analysis further reveals time-dependent interaction stability across all ligand-bound systems (Figure 9). Brigatinib maintains a consistently high number of hydrogen bonds throughout the 500 ns simulation, particularly after ~150 ns, when RMSD stabilization occurs, indicating strong and persistent interactions. Importantly, the screened compounds also demonstrate sustained hydrogen bonding, with SCR00078 and AW01085 showing recurring interaction patterns throughout the simulation, suggesting their ability to maintain stable contacts within the binding pocket. SCR00073 exhibits moderate but dynamic hydrogen bonding, particularly during the 100–200 ns interval, aligning with its stabilization phase (Figure 9). While some fluctuations are observed, such behavior is consistent with flexible binding modes and does not necessarily indicate reduced binding potential, as dynamic interactions can contribute to adaptive ligand accommodation within the active site.

The reduced fluctuations observed across all ligand-bound systems, including the selected compounds, suggest effective modulation of these dynamic regions. Similar to Brigatinib, the screened inhibitors appear to restrict large-scale conformational transitions and reduce overall structural fluctuations relative to the apo system. These observations suggest ligand-associated stabilization of ALK dynamics during the simulation timescale. These stability observations are further supported by MM/PBSA binding free energy calculations, which showed that SCR00078 and AW01085 exhibited more favorable predicted binding free energies than the reference inhibitor Brigatinib, whereas SCR00073 showed comparatively weaker binding (Table 4). Overall, the analysis indicates that while Brigatinib demonstrates strong and consistent stabilization, the screened compounds, particularly SCR00078 and AW01085, exhibit comparable stabilization profiles, whereas SCR00073 maintains a slightly more flexible yet stable binding mode. These findings suggest that the screened compounds effectively stabilize ALK dynamics, with distinct interaction patterns that may contribute to their inhibitory potential.

### 2.10. Principal Component Analysis and Free Energy Landscape Analysis

**Figure 10:**
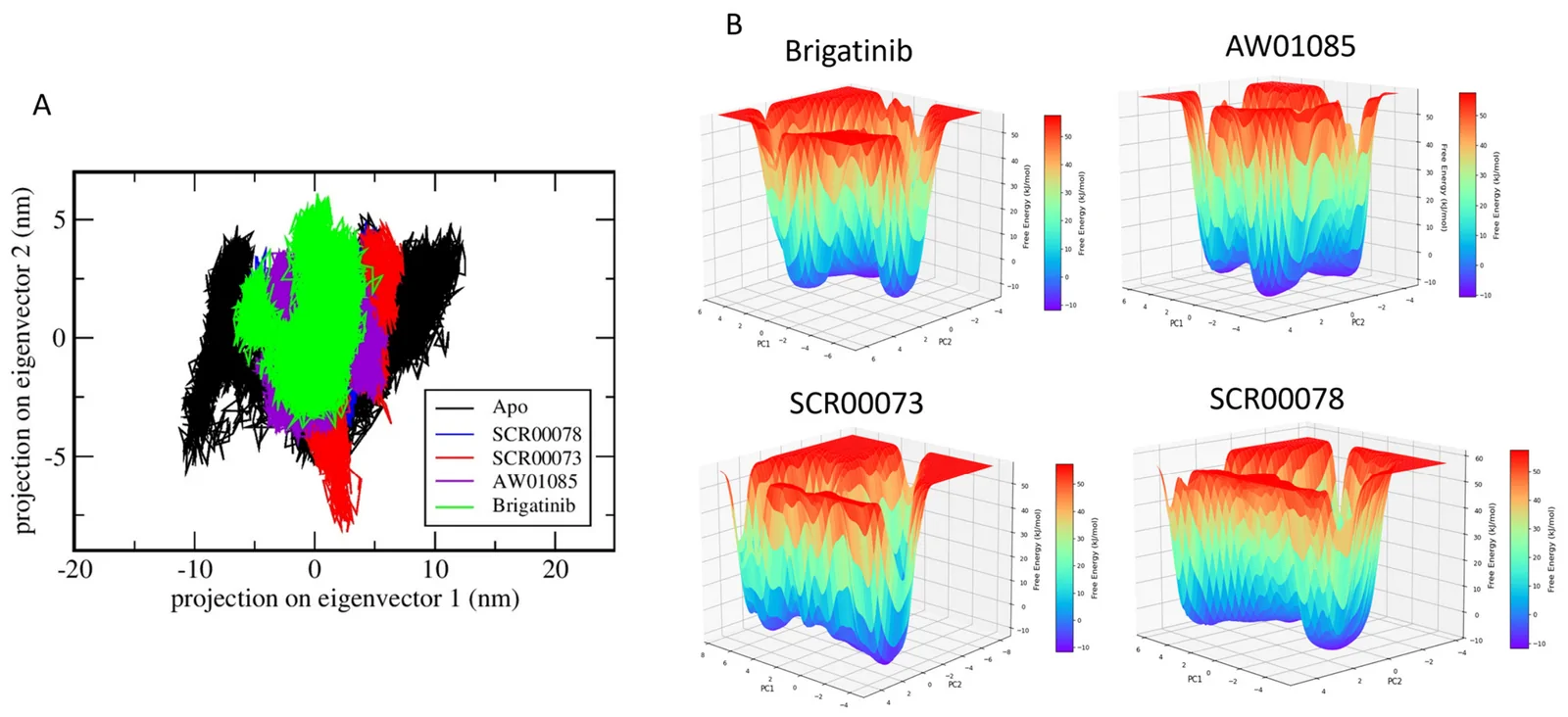
Principal component analysis (PCA) and free energy landscape (FEL) of protein–ligand systems. (**A**) Projection of molecular dynamics trajectories onto the first two principal components (PC1 and PC2), illustrating the conformational space sampled by the apo and ligand-bound systems. (**B**) Free energy landscapes constructed along PC1 and PC2, showing the distribution of low-energy basins and conformational stability of each system.

Principal component analysis (PCA) and free energy landscape (FEL) analyses were conducted to further characterize ligand-induced conformational modulation of ALK (Figure 10). The PCA projection shows that the apo system samples a broad conformational space along both principal components, reflecting the protein’s high intrinsic flexibility and its ability to access multiple conformational states in the absence of ligand binding. In contrast, all ligand-bound systems exhibited substantially reduced conformational sampling, indicating enhanced structural stabilization upon ligand binding (Figure 10). SCR00078 and AW01085 showed relatively compact conformational clusters, whereas SCR00073 retained moderate conformational adaptability (Figure 10). Brigatinib displayed the most restricted conformational distribution, consistent with its known stabilizing effect on ALK. FEL analysis revealed distinct stability profiles among the complexes. SCR00078 displayed two well-defined low-energy basins separated by moderate energy barriers, suggesting a balance between conformational flexibility and stability. SCR00073 exhibited broader low-energy regions, consistent with dynamic adaptability, while AW01085 showed a more rugged landscape characterized by multiple shallow minima, reflecting increased conformational heterogeneity. Brigatinib, included as a reference inhibitor, shows a highly compact cluster consistent with its known stabilizing effect on ALK dynamics, while the clustering behavior of the screened compounds demonstrates comparable trends in restricting protein motion.

The FEL analysis further supports these observations by revealing distinct stability profiles for each inhibitor-bound system (Figure 10B). The SCR00078 complex is characterized by two well-defined low-energy basins separated by a moderate energy barrier (Figure 10B), indicating the presence of dominant metastable states and a balanced combination of structural stability and conformational flexibility. This pattern aligns with protein energy landscape theory, in which biomolecules occupy multiple metastable states separated by energy barriers that govern conformational transitions and functional dynamics. The SCR00073 system exhibits a broader, more continuous low-energy region with fewer distinct minima (Figure 10B), reflecting increased conformational dispersion and lower energy barriers, consistent with its broader PCA distribution and dynamic adaptability. In contrast, the AW01085 complex exhibits a more rugged energy landscape characterized by multiple shallow minima, indicating increased conformational heterogeneity and frequent transitions between structural states. Despite these differences, all screened inhibitors maintain low-energy, accessible conformations, confirming their ability to stabilize ALK relative to the apo state. Collectively, the combined PCA and FEL analyses indicate that the screened inhibitors effectively reduce ALK’s conformational freedom and promote energetically favorable states, though with varying degrees of rigidity and flexibility. SCR00078 demonstrates a favorable balance between structural stability and conformational adaptability, SCR00073 retains moderate flexibility within a stabilized framework, and AW01085 allows greater conformational diversity while still maintaining low-energy states. These findings suggest that, rather than enforcing a single rigid conformation, the screened inhibitors stabilize ALK through distinct dynamic profiles, which may be advantageous for interacting with the kinase’s flexible regions and contributing to their inhibitory potential. Overall, FEL analysis provides an additional dimension for hit prioritization by revealing how ligand binding reshapes the conformational energy landscape of ALK. Unlike conventional stability metrics that primarily quantify structural fluctuations, FEL analysis identifies energetically favorable conformational states and the barriers governing transitions between them. Compounds that stabilize well-defined low-energy basins may be more effective in maintaining inhibitory conformations, whereas highly dispersed energy landscapes can reflect increased conformational heterogeneity. Therefore, the FEL results complement RMSD, RMSF, and MM/PBSA analyses by providing mechanistic insight into ligand-induced conformational regulation and may assist future lead optimization efforts aimed at controlling ALK dynamics.

### 2.11. Structural Uniqueness Analysis of Maybridge Hits

**Figure 11:**
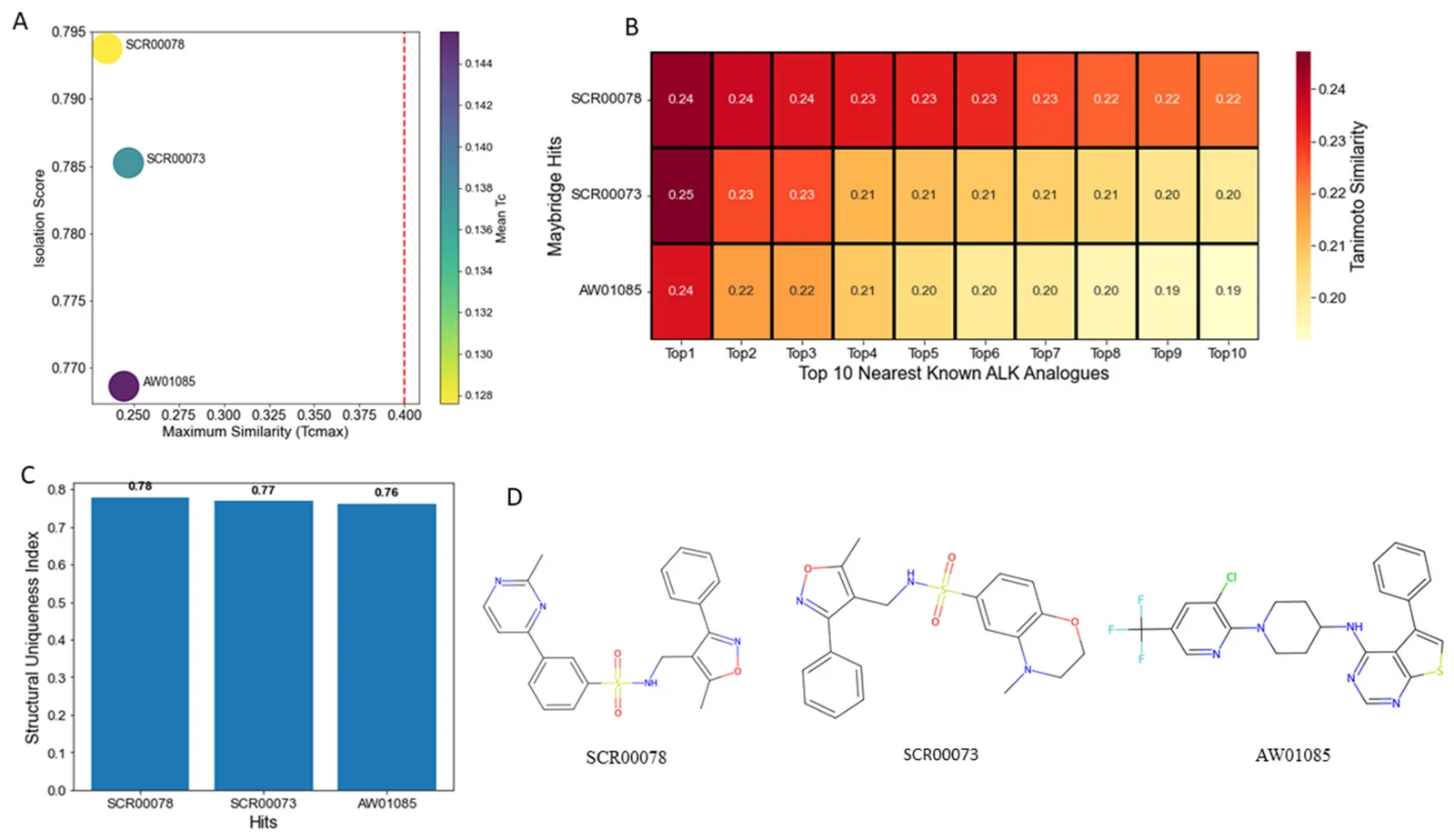
Structural uniqueness analysis of the identified Maybridge hits. (**A**) Novelty–isolation bubble plot showing maximum Tanimoto similarity (Tcmax) and isolation score, where bubble size represents the Structural Uniqueness Index (SUI). (**B**) Heatmap of Top-10 nearest-neighbor (Tanimoto similarity scores against known ALK inhibitors. (**C**) SUI-based ranking of the screened Maybridge hits, highlighting their scaffold novelty and chemical space isolation. (**D**) 2D structure of the compounds.

The structural novelty of the selected Maybridge hits was evaluated using the Structural Uniqueness Index (SUI). The novelty–isolation plot (Figure 11A) showed that all hits possessed very low maximum similarity values (Tcmax ≈ 0.24–0.25), indicating substantial structural divergence from known ALK inhibitors. Among the compounds, SCR00078 exhibited the highest isolation score (~0.794), followed by SCR00073 (~0.785) and AW01085 (~0.769). The large bubble sizes further confirmed high SUI values for all hits. The Top-10 nearest-neighbor heatmap (Figure 11B) demonstrated uniformly low similarity values ranging from ~0.19 to 0.25 across all compounds, confirming minimal overlap with known ALK inhibitor scaffolds. Notably, none of the hits exceeded a Tc value of 0.25, highlighting their occupation of distinct chemical space. The SUI ranking plot (Figure 11C) further supported these findings, with all compounds showing high SUI values (>0.75) and being classified as “Highly Unique.” SCR00078 achieved the highest SUI (~0.78), followed closely by SCR00073 (~0.77) and AW01085 (~0.76). Figure 11D shows the 2D chemical structures of the identified hits. Overall, the analysis indicates that the identified Maybridge hits represent structurally novel ALK inhibitor chemotypes with strong scaffold-hopping potential.

## 3. Discussion

The present study demonstrates the successful integration of machine learning, virtual screening, molecular docking, and molecular dynamics simulations for the identification of potential ALK inhibitors. The combined computational workflow enabled the development of robust predictive models while simultaneously providing mechanistic insights into ligand–ALK interactions and conformational stabilization. Similar integrative AI-assisted drug discovery frameworks have increasingly been applied in kinase inhibitor discovery because they improve hit prioritization and reduce experimental screening costs [3,15]. Among the evaluated machine learning approaches, the ensemble-based RF and XGB models consistently outperformed SVM, particularly when molecular fingerprints were used as input features. This improved performance likely reflects the ability of ensemble algorithms to capture complex nonlinear relationships between molecular substructures and biological activity. Fingerprint-based representations provided superior predictive power compared with traditional 2D descriptors, indicating that topological and substructural information plays a critical role in defining ALK inhibitory activity. Similar observations have been reported in previous QSAR studies where RF and gradient boosting methods demonstrated enhanced performance for kinase inhibitor classification tasks due to their robustness toward high-dimensional chemical descriptors [16]. The reduced predictive performance observed for the moderate-activity class likely reflects overlapping structural characteristics between high- and low-activity compounds. Such behavior is common in multiclass QSAR modeling because moderate compounds often share physicochemical properties with both highly active and weakly active molecules, thereby complicating class separation. Nevertheless, the consistently high AUC values obtained for high- and low-activity classes indicate that the developed models effectively captured meaningful SAR trends relevant to ALK inhibition.

To contextualize the present hits within the broader ALK inhibitor landscape, it is important to note that several approved ALK inhibitors have previously been examined using computational approaches. Representative literature examples are summarized in Supplementary Table S1. Crizotinib has been investigated in ALK resistance models involving L1196M and G1269A mutations, where MD simulations helped explain altered ATP-pocket accommodation and reduced inhibitor stability [17]. Ceritinib resistance has similarly been examined in F1174C and G1202R ALK systems, highlighting mutation-dependent changes in kinase dynamics and energetic stability [18,19], while alectinib has been studied using docking, MD, MM/GBSA, and umbrella sampling to explain G1202R solvent-front resistance [20]. Brigatinib was therefore selected as the principal comparator because it is the co-crystallized inhibitor in the ALK structure used for docking [21], enabling direct re-docking validation and a matched MD/MM-PBSA comparison. Prior structural work has also compared Brigatinib with crizotinib, ceritinib, and alectinib, supporting its relevance as a clinically established ATP-pocket benchmark [22]. More recent computational studies on lorlatinib and compact next-generation ALK inhibitors further reinforce that docking scores alone are insufficient and that ligand-induced conformational stability and free-energy trends are critical for interpreting ALK inhibitor binding [23,24]. In this context, SCR00078, SCR00073, and AW01085 should be viewed as computationally prioritized novel scaffolds whose predicted ALK-binding profiles are benchmarked against Brigatinib and grounded in the established structure-based literature on ALK inhibition.

Chemical space analysis further demonstrated that the training and test datasets occupied overlapping yet structurally diverse chemical regions. PCA revealed substantial overlap between the training and test compounds, indicating that both datasets were sampled from a similar chemical domain and supporting the reliability of model evaluation. In addition, the Tanimoto similarity heatmap confirmed adequate chemical space coverage while highlighting localized clusters of structurally related compounds. The predominance of low similarity values and the broad distribution observed in the PCA space collectively indicate considerable scaffold diversity, which is essential for developing robust and generalizable predictive models [25,26]. The observed intra-scaffold variability additionally highlights the importance of substituent-level optimization in modulating ALK inhibitory potency, consistent with medicinal chemistry studies emphasizing the role of fine structural modifications in kinase inhibitor selectivity [27].

Although the mean accuracy of the Y-scrambled models remained moderate because of the imbalanced multiclass dataset, macro-averaged precision, F1-score, and ROC-AUC declined substantially, with ROC-AUC approaching random classification (~0.5). These findings indicate that the models lost their discriminatory ability after label permutation, supporting that the predictive performance of the original models was not driven by chance correlations. Such validation is considered essential in QSAR studies to ensure model credibility and minimize the risk of chance correlations [28].

The SHAP analysis provided mechanistic interpretability by identifying lipophilicity, electronic distribution, molecular complexity, and surface area as major contributors to ALK inhibitory activity. Descriptors such as BCUT2D_LOGPLOW, SlogP_VSA8, VSA_EState2, and PEOE_VSA1 highlighted the importance of hydrophobic and electrostatic interactions within the ATP-binding cleft of ALK. These findings are consistent with experimentally validated SAR studies of clinically approved ALK inhibitors, including crizotinib, ceritinib, alectinib, brigatinib, and lorlatinib, where optimized lipophilicity and electrostatic complementarity are critical determinants of potency and selectivity [15]. In addition, the significance of BertzCT suggests that moderate molecular complexity favors efficient kinase recognition while minimizing excessive steric hindrance.

Virtual screening of the Maybridge library followed by molecular docking identified three promising compounds, SCR00078, SCR00073, and AW01085, with favorable interaction profiles against the ALK ATP-binding site. All compounds demonstrated conserved interactions with key residues such as Met1189, Leu1122, Val1170, Leu1188, Asp1193, and Arg1241, which are known to play important roles in ATP-competitive kinase inhibition [3,10]. The hydrogen bonding interaction with Met1189 is particularly significant because hinge-region interactions are widely recognized as a defining feature of potent kinase inhibitors [11]. Furthermore, interactions involving Asp1193 suggest engagement with the activation loop/DFG motif region, which plays a major role in kinase regulation and inhibitor selectivity [29]. Although SCR00073 and SCR00078 share the same scaffold, they adopt distinct predicted binding orientations within the ALK binding site. Nevertheless, both compounds retain the conserved hinge interaction with Met1189 and key hydrophobic contacts. Despite their different orientations, they maintain interactions with conserved kinase motifs, including the hinge region and residues surrounding the DFG motif, suggesting that the shared scaffold can adopt alternative binding geometries while preserving essential kinase-binding interactions.

The molecular dynamics simulations further demonstrated that ligand binding significantly reduced ALK conformational flexibility compared with the apo system. The pronounced fluctuations observed in the apo kinase between ~80–200 ns indicate substantial conformational rearrangements within the kinase domain. Such conformational flexibility is commonly observed in protein kinase dynamics and may influence protein function [30,31]. In contrast, ligand-bound systems exhibited earlier equilibration and greater structural stability, indicating that ligand binding effectively constrained large-scale conformational motions. Brigatinib demonstrated the strongest stabilization effect, consistent with its established inhibitory activity, while SCR00078 and AW01085 showed comparable stabilization profiles. SCR00073 retained a slightly more flexible yet stable binding mode, suggesting adaptive accommodation within the binding pocket rather than destabilization. Hydrogen bond analysis further supported these findings by showing persistent ligand–protein interactions throughout the simulations, particularly for SCR00078 and AW01085. Stable hydrogen bonding interactions are known to contribute substantially to kinase inhibitor affinity and ATP-competitive residence time [32]. The reduction in RMSF values observed upon ligand binding suggests decreased flexibility in several regions of the kinase domain relative to the apo system. Some of these regions spatially overlap with known regulatory elements, including the activation loop and glycine-rich P-loop, which play important roles in kinase regulation [33,34].

The PCA projection shows that the apo system samples a broad conformational space along both principal components, reflecting high intrinsic flexibility and the ability of the protein to access multiple conformational states in the absence of ligand binding, a behavior commonly observed in ligand-free protein systems [35]. SCR00078 and AW01085 exhibited relatively compact conformational clustering, suggesting strong stabilization of the kinase domain. In contrast, SCR00073 retained greater conformational adaptability. Such ligand-induced suppression of global protein motions and enhanced structural stabilization has been widely reported in molecular dynamics studies of kinase–inhibitor complexes [36]. While their overall flexibility is lower than that of the apo system, it retains some conformational adaptability, suggesting a stable yet dynamic binding behavior. This pattern is consistent with the conformational selection and induced-fit mechanisms often seen in protein–ligand interactions. In such cases, ligands not only recognize pre-existing conformations but also induce local structural changes during binding [37]. FEL analysis further demonstrated that all screened compounds stabilized energetically favorable conformations relative to the apo state. From a drug discovery perspective, these results suggest that the identified hits not only bind to ALK but also modulate its conformational landscape. Such information is valuable during hit-to-lead optimization because compounds capable of stabilizing favorable conformational ensembles may exhibit improved target engagement. The presence of multiple metastable states and shallow minima, particularly in AW01085, reflects a rugged energy landscape characteristic of flexible protein–ligand systems [38]. The structural uniqueness analysis demonstrated that all identified hits possessed low Tanimoto similarity values (<0.30) relative to known ALK inhibitors, indicating substantial scaffold novelty and distinct chemical space occupancy. Moreover, all compounds exhibited high Structural Uniqueness Index (SUI) values (>0.70), classifying them as highly unique chemotypes. The heterocyclic and halogenated frameworks observed in the identified hits further support their structural diversity and highlight their potential as scaffold-hopping candidates for novel ALK inhibitor discovery.

Therefore, the integrated computational strategy employed in this study successfully prioritized structurally diverse candidate ALK inhibitor scaffolds for future experimental evaluation. The combined machine learning, docking, and molecular dynamics analyses suggest that SCR00078, SCR00073, and AW01085 represent promising scaffolds for further lead optimization and experimental validation. Importantly, the distinct conformational stabilization patterns observed for these compounds may provide opportunities to design inhibitors that effectively target the dynamic regulatory mechanisms underlying ALK activity.

## 4. Materials and Methods

### 4.1. Data Curation

Chemicals with activity toward ALK were retrieved from the ChEMBL database (version 36, released in July 2025) using the target identifier CHEMBL4247 (ALK tyrosine kinase receptor). The dataset comprised experimentally determined IC_50_ values obtained from single-protein assays associated with *Homo sapiens* ALK targets. IC_50_ was used as the activity endpoint, and activity values reported in nanomolar (nM) units were used for compound classification. Data curation included the removal of invalid structures, duplicate compounds, and ambiguous activity records containing qualifiers such as “>”, “<”, “≤”, and “≥”. Multiple activity measurements for the same compound were consolidated by retaining the least potent IC_50_ value. All molecules were standardized by converting them into canonical SMILES representations and removing salts or disconnected fragments where applicable. The standardized molecular structures provided by ChEMBL were used directly for descriptor and fingerprint calculation using PaDEL-Descriptor. No additional tautomer enumeration or protonation-state modification was performed during data preparation, and force-field-based atomic charge assignment was not required for descriptor calculation. A total of 1489 unique compounds were retained for model development. For multiclass modeling, compounds with IC_50_ ≤ 0.1 μM (pIC_50_ ≥ 7.0) were classified as High affinity (*n* = 1005), compounds with IC_50_ > 0.1 μM and ≤1 μM (6.0 ≤ pIC_50_ < 7.0) were classified as Moderate affinity (*n* = 318), and compounds with IC_50_ > 1 μM (pIC_50_ < 6.0) were classified as Low affinity (*n* = 166). Finally, the dataset was randomly divided into training and test sets using an 80:20 ratio.

### 4.2. Model Building

Machine learning methods were employed as the artificial intelligence component of the virtual screening workflow to predict ALK inhibitor activity and prioritize compounds for downstream analyses. Three machine learning algorithms, SVM [39], Random Forest (RF) [40], and Extreme Gradient Boosting (XGBoost) [41], were utilized for model construction. The SVM model employed a radial basis function (RBF) kernel with a penalty parameter (C) of 8.0. For the Random Forest model, 500 decision trees were used with the optimized mtry value. Both binary and multiclass classification models were developed. The multiclass classification was performed using the One-vs-All (OvA) strategy. To address data imbalance, the class_weight = ‘balanced’ parameter from Scikit-learn was applied. This approach automatically adjusts the weights of each class in inverse proportion to their frequencies, thereby assigning higher weights to minority classes and lower weights to majority classes. Consequently, misclassifications of minority classes are penalized more heavily, improving model fairness and robustness.

### 4.3. Model Validation

Model performance was assessed on the independent test set. For multiclass model evaluation, Precision-Macro, Recall-Macro, and F1-Macro metrics were calculated. These metrics are particularly suitable for imbalanced datasets, as they give equal weight to each class irrespective of its frequency, ensuring a more balanced assessment of model performance [42,43]. Additionally, model quality was further evaluated using Receiver Operating Characteristic (ROC) curves and the corresponding Area Under the Curve (AUC) values, which provide insight into the discriminatory power of the developed models [44,45]. The statistical performance indicators for each model are summarized in Table 1.

### 4.4. Y-Scrambling

The robustness and reliability of the developed models were assessed using the Y-scrambling technique. In this approach, the target variable values in the training dataset were randomly permuted while keeping the descriptor matrix unchanged [46]. Subsequently, new models were generated using the same modeling parameters as those employed for the original models. Each randomized model produced an accuracy estimate, which was compared with that of the original, unshuffled model. Consistently lower predictive performance from the randomized models indicates that the original model’s predictive power is not due to random correlations, thereby confirming its statistical validity. In this study, 100 Y-scrambling iterations were performed using 50% of the target class data, ensuring a comprehensive evaluation of model robustness and the absence of chance correlations.

### 4.5. Applicability Domain

To ensure the reliability of model predictions and avoid extrapolation beyond the learned chemical space, the Applicability Domain (AD) of the developed model was assessed using a similarity-based approach [47,48]. The AD defines the subset of chemical space where the model’s predictions are considered trustworthy, thereby identifying compounds structurally similar to those used during training. A similarity threshold of 0.5 was employed to define the model’s applicability domain. Compounds with a maximum similarity value greater than or equal to this threshold were considered to be within the AD, indicating that their structural features were adequately represented in the model’s training space. Conversely, compounds below this threshold were regarded as outside the AD, and their predictions may be less reliable due to insufficient structural representation. The distribution of Tanimoto similarity values across different activity classes was visualized through histograms, providing insight into the chemical coverage of each class. A vertical red dashed line in the plot denotes the AD threshold, distinguishing well-represented compounds from potential outliers. The fraction of compounds residing within the defined AD was further summarized for each class, offering a quantitative measure of chemical space coverage. This similarity-based analysis thus confirms that the majority of compounds fall within the model’s defined applicability domain, ensuring the reliability and interpretability of the predictive outcomes.

### 4.6. Scaffolds Analysis

We performed a Bemis–Murcko scaffold analysis on our compound dataset to extract core molecular frameworks (i.e., ring systems plus linkers, excluding terminal substituents) [49]. Scaffold extraction and enumeration were performed using standard cheminformatics procedures, and the resulting scaffolds were grouped and ranked by frequency of occurrence in the dataset. To assess scaffold distribution across activity classes, the frequency of each scaffold was calculated separately across different activity classes. Scaffolds exhibiting higher representation in the high-activity class were considered enriched chemotypes and were prioritized for further structure–activity relationship analysis. Scaffolds showing higher relative occurrence in active compounds were identified as privileged scaffolds, consistent with established definitions in medicinal chemistry [50]. To further prioritize scaffolds, an enrichment score was calculated by integrating scaffold prevalence within the dataset with the average biological activity of compounds associated with each scaffold. For each scaffold, the scaffold frequency (i.e., the number of compounds containing the scaffold), mean pIC50 value, and standard deviation of pIC_50_ were calculated. The Scaffold Enrichment Score was subsequently used to identify scaffolds that were both highly represented and associated with strong biological activity. Scaffolds represented by fewer than five compounds were excluded from the enrichment analysis, and the remaining scaffolds were ranked according to their enrichment scores for visualization and comparative analysis.

### 4.7. Library Screening and Molecular Docking

The best-performing predictive models were used for virtual screening of the Maybridge compound library (Maybridge, https://www.maybridge.com), which contains approximately 51,000 molecules. Compounds predicted to fall within the chemical space of active compounds were further filtered using applicability domain analysis.

### 4.8. Molecular Docking

The three-dimensional structure of human ALK (PDB ID: 6MX8) [21] was obtained from the RCSB Protein Data Bank [51] and prepared using Discovery Studio Visualizer by removing water molecules and heteroatoms. The selected ligands were energy-minimized using the CHARMm force field and subsequently converted into PDBQT format using PyRx [52]. Molecular docking was then performed using AutoDock Vina v1.2.0 [46], which utilizes an empirical scoring function to estimate ligand–protein binding free energies based on a hybrid knowledge-based and force-field-inspired scoring approach. The active site was defined based on the position of the co-crystallized ligand, and a grid box was generated with center coordinates set to X = 4.310375, Y = 20.935700, and Z = 6.695075, using default docking parameters. The resulting docking poses and predicted binding affinities were recorded, and the final interaction profiles, including hydrogen bonding and hydrophobic interactions, were analyzed and visualized in detail. Docking validation was performed by redocking the co-crystallized inhibitor (Brigatinib) into the ALK ATP-binding pocket, confirming accurate reproduction of the experimental binding pose.

### 4.9. Physicochemical and ADMET Profiling (SwissADME and ADMET-AI)

Physicochemical and in silico ADMET profiling was performed for the three shortlisted hits. Canonical SMILES were analyzed using SwissADME [53] to compute key physicochemical descriptors and drug-likeness/structural alert filters (Lipinski/Veber, PAINS, Brenk). ADMET-AI [54] was used to predict pharmacokinetic and safety-related endpoints (e.g., absorption/distribution indicators, CYP liabilities, and toxicity flags such as hERG, AMES, and DILI). Outputs from both tools were summarized as supportive in silico evidence to flag potential liabilities for downstream optimization.

### 4.10. Molecular Dynamics Simulations

Molecular dynamics (MD) simulations were performed in GROMACS (version 2022) [55] using the CHARMM27 all-atom force field for the protein [56]. Ligand parameters/topologies were generated using SwissParam to ensure CHARMM compatibility [57]. Each system (apo ALK and ALK–ligand complexes) was solvated with TIP3P water in a cubic periodic box, maintaining a minimum distance of 1.0 nm between any protein atom and the box edge. Counterions were added to neutralize the system, and 0.15 M NaCl was used to mimic physiological ionic strength. After steepest-descent energy minimization, systems were equilibrated under NVT followed by NPT conditions. Long-range electrostatics were treated with Particle Mesh Ewald (PME), and all bonds involving hydrogen atoms were constrained using LINCS, allowing a 2-fs integration time step. Production MD simulations were run for 500 ns for each system under periodic boundary conditions.

### 4.11. Trajectory Processing and Stability Analyses

Trajectories were de-imaged, centered, and least-squares fitted to protein backbone heavy atoms prior to analysis. Structural stability and flexibility were assessed using standard GROMACS tools: Cα-RMSD, per-residue RMSF, radius of gyration, and protein–ligand hydrogen bonds. Hydrogen bonds were defined using a donor–acceptor distance cutoff of 0.35 nm (3.5 Å) and a minimum angle of 135°.

### 4.12. Principal Component Analysis and Free Energy Landscape

Essential dynamics were analyzed by principal component analysis (PCA) using the covariance matrix of backbone positional fluctuations, and dominant motions were characterized by projecting trajectories onto the first two principal components (PC1 and PC2). The cumulative variance explained by the leading modes was recorded. Free energy landscapes (FELs) were constructed on the PC1–PC2 plane at 300 K to identify low-energy basins and compare conformational sampling between apo and ligand-bound systems.

### 4.13. MM/PBSA Binding Free Energy Calculations

End-state binding free energies (ΔG_bind) were estimated using the MM/PBSA approach with a single-trajectory protocol. Poisson–Boltzmann (PB) calculations [58] were performed using the internal PBSA solver in sander on 5001 frames extracted from the equilibrated portion of each 500 ns trajectory [59,60]. Energy components were decomposed into van der Waals (ΔE_vdW), electrostatic (ΔE_elec), polar solvation (ΔG_PB), and nonpolar solvation terms (ΔG_nonpolar; reported as ΔE_NPOLAR, with the dispersion term ΔE_DISPER included when enabled). Total binding free energy was computed as ΔG_total = ΔE_vdW + ΔE_elec + ΔG_PB + ΔG_nonpolar, and results are reported as mean ± SEM across frames.

### 4.14. Structural Uniqueness Index (SUI) Calculation

To quantify the structural separation of query compounds from a reference chemical space, an Isolation Score (IS) was defined based on molecular fingerprint similarity distributions. The Tanimoto similarity between two molecular fingerprints A and B was calculated as [61,62]:

$$T_{c}\left(A,B\right)=\frac{A\cap B}{A+B-A\cap B}$$

For each hit compound, the maximum similarity score (Tcmax) against the reference dataset (train set) was determined. The mean similarity of the top nearest neighbours was then calculated as

$${TopN}_{mean}=\frac{1}{N}\sum_{i=1}^{N}{Tc}_{i}$$

where ${Tc}_{i}$ is the Tanimoto similarity of the i^th^ nearest neighbor; and N represents the number of nearest neighbours considered (N = 10).

The isolation score was computed as

$$Isolation=1-{TopN}_{mean}$$

Finally, the Structural Uniqueness Index (SUI) was calculated using

$$SUI=\sqrt{\left(1-{Tc}_{max})(1-{TopN}_{mean}\right)}$$

In this study, we developed an AI-assisted virtual screening workflow based on multiclass machine learning models for ALK inhibitor discovery and integrated it with scaffold analysis and structure-based simulations to prioritize novel candidate ALK inhibitors from the Maybridge library.

## 5. Conclusions

In this study, we developed multiclass machine learning models for ALK inhibitors and integrated them with scaffold analysis and structure-based simulations to computationally prioritize novel candidate ALK inhibitors from the Maybridge library. The validated models achieved robust multiclass performance, and applicability-domain filtering supported reliable prioritization of external-library candidates. Molecular docking identified three candidate hits (SCR00078, SCR00073, and AW01085) that exhibited comparable predicted binding modes and interaction profiles. These candidates were subsequently prioritized for 500 ns molecular dynamics simulations. Trajectory analyses indicated stable complex formation for all screened ligands with reduced conformational dispersion relative to the apo system. MM/PBSA calculations further ranked SCR00078 and AW01085 as the most favorable binders, with predicted ΔGtotal values exceeding those of the reference inhibitor Brigatinib, whereas SCR00073 showed comparatively weaker binding. Overall, SCR00078 and AW01085 emerged as the most promising ALK inhibitor candidates based on the integrated machine learning and structure-based analyses. However, the findings presented in this study are based on computational analyses and do not constitute experimental validation of ALK inhibition. Therefore, the identified compounds should be regarded as potential ALK inhibitor candidates. Further studies will be required to confirm their inhibitory activity, characterize their selectivity, and assess their activity against clinically relevant ALK resistance variants.

## Figures and Tables

**Table 1:** Performance evaluation metrics of validated multiclass models on the test set.

Descriptors Set	Methods	Metrices				ROC-AUC		
		Accuracy	Precision [Macro]	Recall [Macro]	FI [Macro]	High	Moderate	Low
2D Descriptors	SVM	0.79	0.71	0.64	0.66	0.89	0.75	0.89
	RF	0.75	0.66	0.62	0.63	0.89	0.75	0.89
	XGB	0.77	0.68	0.65	0.67	0.89	0.77	0.90
Fingerprints (MACCS and ECFP4)	SVM	0.77	0.68	0.64	0.65	0.90	0.76	0.91
	RF	0.78	0.69	0.68	0.66	0.91	0.74	0.93
	XGB	0.75	0.67	0.66	0.66	0.90	0.75	0.94

**Table 2:** Performance metrics of Y-scrambled models (mean ± SD).

Method	Descriptor	Accuracy ± SD	Precision ± SD	F1 ± SD	ROC-AUC ± SD		
					High	Moderate	Low
SVM	2D	0.36 ± 0.05	0.48 ± 0.048	0.40 ± 0.055	0.48 ± 0.056	0.48 ± 0.051	0.49 ± 0.043
	Fingerprint	0.50 ± 0.029	0.47 ± 0.027	0.48 ± 0.26	0.50 ± 0.051	0.49 ± 0.050	0.50 ± 0.052
Random Forest	2D	0.61 ± 0.014	0.48 ± 0.032	0.52± 0.014	0.44 ± 0.052	0.46 ± 0.050	0.44 ± 0.057
	Fingerprint	0.60 ± 0.016	0.49 ± 0.032	0.52 ± 0.016	0.50 ± 0.046	0.51 ± 0.050	0.48 ± 0.050
XGBoost	2D	0.59 ±0.0218	0.493 ± 0.030	0.52 ± 0.037	0.45 ± 0.043	0.47 ± 0.047	0.42 ± 0.050
	Fingerprint	0.57 ± 0.019	0.48 ± 0.024	0.51 ± 0.018	0.43 ± 0.040	0.45 ± 0.039	0.38 ± 0.056

**Table 3:** Compact consensus ADMET summary for three shortlisted compounds (SwissADME + ADMET-AI).

Compound	Drug-Likeness (SwissADME)	GI Absorption	BBB	P-gp	CYP Inhibition (Predicted)	hERG	AMES	DILI
AW01085	Lipinski: 1 violation; Veber: Pass; PAINS/Brenk: 0/0	High	Yes	Yes	1A2, 2C19, 2C9, 3A4	High	Low	High
SCR00073	Lipinski: Pass; Veber: Pass; PAINS/Brenk: 0/0	High	No	Yes	1A2, 2C19, 2C9, 3A4	High	Low	High
SCR00078	Lipinski: Pass; Veber: Pass; PAINS/Brenk: 0/0	High	No	Yes	1A2, 2C19, 2C9, 3A4	High	Low	High

AW01085 shows higher lipophilicity (Lipinski violation) and predicted BBB permeation. All three compounds were flagged for potential hERG and DILI liabilities and multi-CYP inhibition propensity, which should be considered during optimization and experimental validation.

**Table 4:** MM/PBSA ligand–ALK binding free energy components (mean ± SEM; kcal/mol). Calculations were performed using 5001 frames (Poisson–Boltzmann calculations using the internal PBSA solver in sander).

Complex	ΔE_vdW	ΔE_elec	ΔG_PB	ΔE_NPOLAR	ΔG_gas	ΔG_solv	ΔG_total
Brigatinib (Control)	−25.38 ± 3.97	−252.65 ± 23.69	260.47 ± 22.00	−3.16 ± 0.31	−278.03 ± 23.21	257.31 ± 21.98	−20.72 ± 3.65
SCR00078	−17.54 ± 3.37	−380.76 ± 19.66	372.87 ± 18.33	−3.35 ± 0.18	−398.31 ± 19.28	369.52 ± 18.27	−28.78 ± 3.11
SCR00073	−31.63 ± 11.40	−191.13 ± 37.86	209.42 ± 24.81	−3.72 ± 0.81	−222.76 ± 28.90	205.70 ± 24.34	−17.06 ± 6.43
AW01085	−17.34 ± 3.83	−417.97 ± 43.79	414.59 ± 41.56	−2.56 ± 0.41	−435.31 ± 45.27	412.03 ± 41.26	−23.27 ± 5.45

## Data Availability

All data analyzed during this study are included in the manuscript and its Supplementary Materials. Further inquiries can be directed to the corresponding author(s).

## Supplementary Materials

The following supporting information can be downloaded at: https://www.mdpi.com/article/10.3390/ph19081209/s1, Figure S1: Flowchart showing steps used for virtual screening of chemical library; Table S1: Literature context for approved and clinically relevant ALK inhibitors in structure-based computational studies.
