# Supplementary material for: Multiclass Machine Learning-Based Discovery of Novel Scaffold Inhibitors Targeting ALK
Source: Pharmaceuticals (Basel). 2026 Aug 1;19(8):1209. doi: 10.3390/ph19081209 (PMC13517032; doi:10.3390/ph19081209)
Supplement: Supplementary file 1 [file pharmaceuticals-19-01209-s001.zip › pharmaceuticals-4360905-supplementary.pdf]

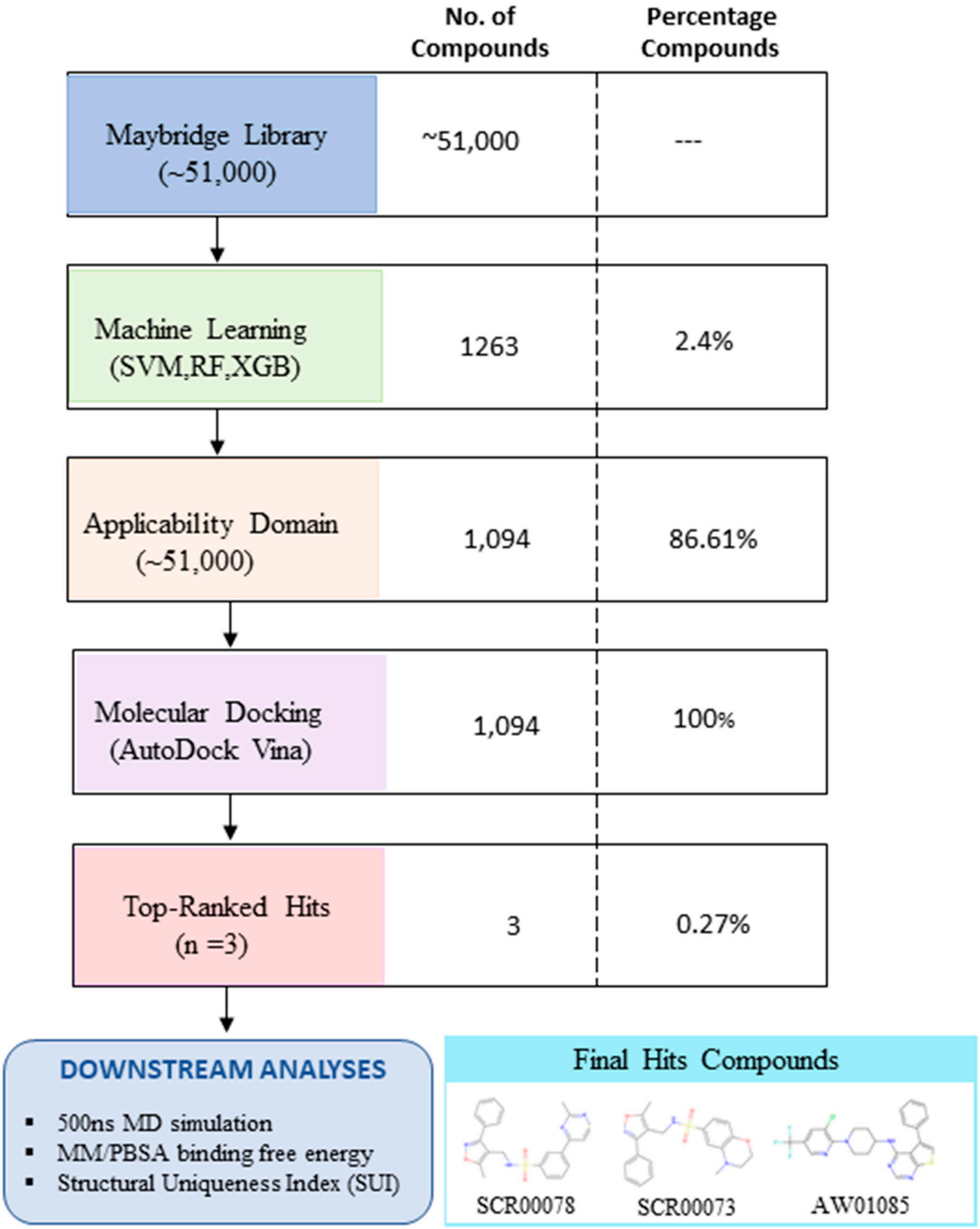

Supplementary Figure S1. Flowchart showing steps used for virtual screening of chemical library.

Supplementary Table S1. Literature context for approved and clinically relevant ALK inhibitors in structure-based computational studies.

Inhibitor	Context	Computational relevance	Relevance to this study	References
Crizotinib	First-generation ALK inhibitor	Docking/MD studies examined L1196M and G1269A resistance.	Shows that ATP-pocket mutations can alter inhibitor stability and pocket accommodation.	[17]
Ceritinib	Second-generation ALK inhibitor	MD studies examined F1174C and G1202R resistance.	Supports dynamic interpretation of ALK inhibitor binding and mutation-dependent energetic stability.	[18,19]
Alectinib	Second-generation ALK inhibitor	Docking, MD, MM/GBSA and umbrella sampling were used to study G1202R solvent-front resistance.	Reinforces the importance of solvent-front effects, binding stability and free-energy analysis.	[20]
Brigatinib	Clinical comparator in this study	Co-crystallized ALK ligand; structurally compared with other ALK inhibitors.	Provides the matched benchmark for re-docking, MD and MM/PBSA comparison.	[21,22,51]
Lorlatinib / next-generation inhibitors	Later-generation and compact ALK inhibitors	Computational studies emphasize compact scaffolds, resistant-pocket accommodation and conformational dynamics.	Supports evaluating novel scaffolds beyond docking score alone.	[23,24]

Note: Reference numbers correspond to the main manuscript reference list.
